# Optimal Dummy Pattern Design Method for PWB Warpage Control Using the Human-Based Genetic Algorithm

**DOI:** 10.3390/mi11090807

**Published:** 2020-08-25

**Authors:** Sun Kyoung Kim, Sang-Hyuk Lee

**Affiliations:** Department of Mechanical System Design Engineering, Seoul National University of Science and Technology, Seoul 02421, Korea; sanghyuk.lee@seoultech.ac.kr

**Keywords:** printed wiring board (PWB), warpage, genetic algorithm (GA), electronic package, simulation

## Abstract

In this work, a method that minimizes printed wiring board (PWB) warpage by dummy pattern design is proposed. This work suggests that dummy patterns are placed on a preset discretized location in the PWB to reduce the warpage. On each discretized candidate area, the dummy pattern can be set or unset. The warpage is numerically simulated based on direct modeling of the as-is PWB patterns to evaluate the warpage alongside the dummy pattern design set. The optimal pattern that minimizes warpage is determined using the human-based genetic algorithm where the objective function is evaluated by the structural simulation. The optimization method is realized in a spreadsheet that allows scripting language with which the input and output files of the simulation tool can be modified and read. Two different cases have been tested and the results show that the method can determine the optimal dummy patterns. The measured and simulated deflections agree well with each other. Moreover, it has been shown that certain dummy pattern designs that should reduce the warpage can be sought by the optimization.

## 1. Introduction

**Figure 1:**
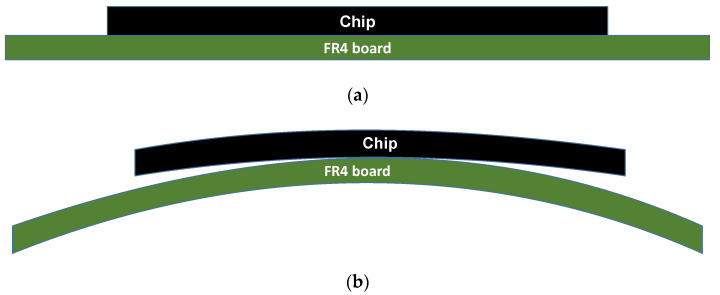
Printed wiring board (PWB) and chip deformation (**a**) at a processing temperature in stress-free conditions and (**b**) at room temperature after cooling.

Warpage of the printed wiring board (PWB) raises serious quality issues including assemblage and long-term reliability [1,2,3,4]. It also incurs component misalignments and interconnect failures which can result in the failure of the entire assembly [2,5]. The PWB at a processing or reflow temperature can be stress-free and would not show any significant warpage as shown in Figure 1a. However, it will warp while cooling to room temperature, resulting in high interlayer stress and potential interconnect failure as illustrated in Figure 1b [6]. When heating up for the reflow process followed by cooling down to room temperature, the package experiences a dramatic difference in thermal strains depending on the location and materials during the cooling down. If such strains are restrained by mechanical joining, the corresponding residual stress will remain. 

Furthermore, warpage in the bare PWB may induce cracks and delamination during and after assembly. The PWB substrate is getting thinner as the package density increases. In the meantime, the process and operating temperatures are getting higher. To comply with such demands, the PWB industry has to further suppress the warpage by improving the design and fabrication process. However, the curing process requires elevated temperatures, which inevitably amplify the non-uniformity of the material strength inherent in the design. Sometimes engineers are tempted to rectify the warpages by external forces but that can result in another type of failure due to the residual stress. To control the warpage a PWB manufacturer does not have many options to try. Since the PWB designs are usually optimized in view of circuit performance, the industry convention does not allow modification of the circuit pattern to improve manufacturability or mechanical performance. Moreover, the PWB industry is quite conservative in changing process parameters and methods. As a result, the placement of a copper pattern on a vacant area where a circuit pattern is absent can be a viable method since the copper pattern can alter the mechanical characteristics of the board. Such copper patterns are called dummy patterns and have been widely utilized to control the warpage. Changing dummy patterns does not affect the circuit performance and is one of the best ways the PWB industry can select [7,8]. There are some other techniques that can remedy warpage. Those methods can be classified into three types. First, thermal annealing can relax the stress present in the warpage. Second, a thermo-mechanical leveling method can alleviate existing warpage. Third, a mechanical stiffening method can reduce the warpage. All these are widely used in practice, but they increase manufacturing costs and impact reliability as well as flexibility [9]. In many cases, even with these techniques, warpage corrections are limited.

This study proposes a method that can systematically set the dummy pattern in the PWB. This method requires the initial setup of candidate areas for the dummy pattern. The candidate areas are two-dimensional simply connected domains where the copper cladding can be constituted without influence on the main circuit pattern. Each candidate can be set or unset to improve the warpage characteristics. That is, if the copper pattern in a specific candidate area decreases the warpage, that area is set, and otherwise, it is unset. The procedure based on the finite element method has been well stated in a published patent [10] and its performance has been verified for a double-sided PWB [11]. Since the publication of such works, the PWB industry is still coping with the problem by trial and error. However, what is promising is that the industry is now widely using simulation tools to predict warpage. Nevertheless, the simulation process is quite laborious and resource intensive in terms of expertise and knowledge availability. In this work, to overcome such a situation, the optimization method has been improved to enhance its efficiency. In addition, the method has been applied to a multilayer package. In this paper, first, the simulation and the optimization methods are described sequentially. Then, the methods are applied to the two warpage problems.

## 2. Methods

### 2.1. Overall Approach

The aforementioned candidate areas should be determined first. Usually, there are limited locations available to set the dummy pattern. If there are only two discrete areas selected for the candidates, the possible ways of setting dummy patterns are only four, which are setting both, either of them and none. However, if ten discrete areas are selected, there exist 2^10^ ways of setting the dummy pattern. The evaluation of warpage demands significant computational cost since the structure should be discretized into many small elements. Thus, it is beneficial to reduce the number of evaluations. In other words, the candidate areas can be further divided into many discrete areas.

**Figure 2:**
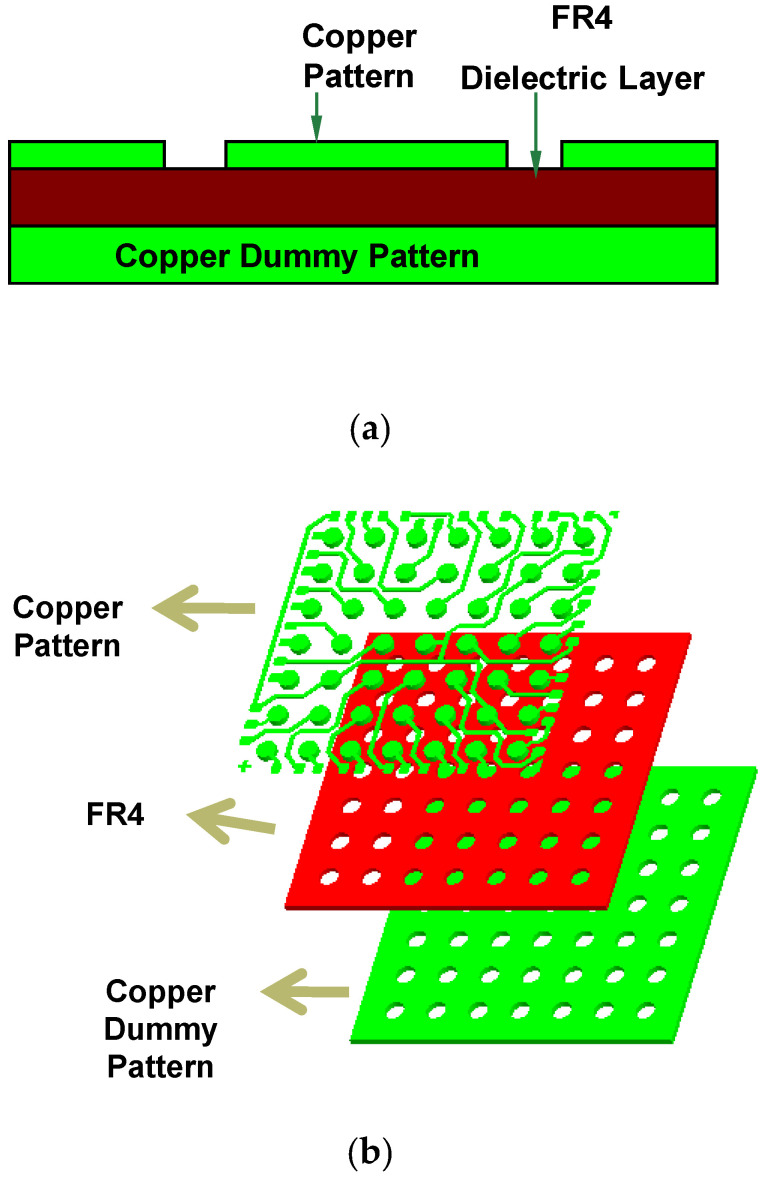
(**a**) Conceptual structure of PWB (**b**) construction of the three-dimensional MCAD model.

The conventional PWB for electronic packages are comprised of the stacked dielectric (FR4) and copper pattern layers with the solder resist (SR) on the outermost layers. Complex modern packages are bound to be multilayered. This work will show two different cases: the first case investigates a double sided PWB with a middle dielectric layer as in Figure 2a and the second case looks at a multilayered PWB.

To save design time, both the evaluation by simulation and the optimization of the pattern setting should be conducted in an efficient way. This work adopts the human-based genetic algorithm for the pattern optimization and the shell-solid hybrid model for the finite element (FE) simulation [12,13,14,15]. The details are presented in the coming sections.

### 2.2. Simulation Model

The simulation problem in this work is a three-dimensional thermo-elasticity problem of which deformation is mostly concentrated in the out-of-plane (thickness) direction [13]. Here arises a major challenge. It is extremely difficult to generate the geometric model owing to the complexity of the copper patterns. To alleviate this difficulty, homogenization of the complex copper layer has been conducted in existing works [16,17,18,19]. This method imposes equivalent material properties on the domain instead of modeling the cladding as it is. However, in many cases, this can be oversimplification since such an approximation by homogenization cannot take pattern continuity into consideration. It is reported that the pattern continuity affects PWB warpage as well as its direction and width [8,14]. It is quite evident that continuation of normal and shear-stress flows dramatically affect overall rigidity. Moreover, recent advances in computational capability allow this direct simulation. In this work, the entire cladding pattern is modeled to realistically simulate the warpage. Based on the proposed three-dimensional model, the warpage simulation is repeatedly conducted to find the optimal dummy pattern.

A full three-dimensional mechanical CAD (MCAD) solid model had to be constructed from the electronic art work data, which was contained in a two-dimensional electronic CAD (ECAD) file [20,21]. Construction by such a conversion is not straightforward at all. The ECAD data comprise many inhomogeneous entities including diverse lines and areas, which are not easily rearranged in the MCAD. To successfully accomplish this conversion task, several functions in the CAD tools have been utilized. From an ECAD tool, closed polylines were created. Next, they were converted to three-dimensional surface areas and then finally to three-dimensional thin solids in an MCAD tool. Figure 2b shows the construction method of the MCAD model. It should be noted that the solder resister layers are neglected in this model, as they do not dominate the PWB warpage.

A warpage simulation requires the solution of a partial differential equation that describes the mechanical deformation. The stress components should be in balance. The normal stresses, $\sigma_{x}$, $\sigma_{y}$, $\sigma_{z}$ and the shear stresses, $\tau_{xy}=\tau_{yx}$, $\tau_{yz}=\tau_{zy}$, $\tau_{zx}=\tau_{xz}$, should satisfy [14,22,23]

(1a) $$\frac{\partial \sigma_{x}}{\partial x}+\frac{\partial \tau_{xy}}{\partial y}+\frac{\partial \tau_{xz}}{\partial z}+f_{x}=0$$

(1b) $$\frac{\partial \tau_{xy}}{\partial x}+\frac{\partial \sigma_{y}}{\partial y}+\frac{\partial \tau_{yz}}{\partial z}+f_{y}=0$$

(1c) $$\frac{\partial \tau_{zx}}{\partial x}+\frac{\partial \tau_{yz}}{\partial y}+\frac{\partial \sigma_{z}}{\partial z}+f_{z}=0$$

where the body forces are specified as $f_{x}=f_{y}=0$ and $f_{z}=-\rho g$. The gravitational acceleration, *g*, and the density, *ρ*, should be given. To solve the above problem, a constitutive relation should be imposed. The PWB board can be considered an orthotropic material whereas other packaging materials such as copper, gold and silicon are regarded as isotropic ones. Assuming a linear Hookean elastic deformation of an orthotropic material, the stress and strain under a thermal change of ΔT should obey the following relationship [23]:

(2a) $$\varepsilon_{x}=\frac{\sigma_{x}}{E_{x}}-\frac{\nu_{yx}\sigma_{y}}{E_{y}}-\frac{\nu_{zx}\sigma_{z}}{E_{z}}+\alpha_{x}\Delta T$$

(2b) $$\varepsilon_{y}=-\frac{\nu_{xy}\sigma_{x}}{E_{x}}+\frac{\sigma_{y}}{E_{y}}-\frac{\nu_{zx}\sigma_{z}}{E_{z}}+\alpha_{y}\Delta T$$

(2c) $$\varepsilon_{z}=-\frac{\nu_{xz}\sigma_{x}}{E_{x}}-\frac{\nu_{yz}\sigma_{y}}{E_{y}}+\frac{\sigma_{z}}{E_{z}}+\alpha_{z}\Delta T$$

(2d) $$\gamma_{yz}=\frac{\tau_{yz}}{G_{yz}}$$

(2e) $$\gamma_{zx}=\frac{\tau_{zx}}{G_{zx}}$$

(2f) $$\gamma_{xy}=\frac{\tau_{xy}}{G_{xy}}$$

where the Young’s moduli, $E_{x}$, $E_{y}$, $E_{z}$, the shear moduli, $G_{yz}$, $G_{zx}$, $G_{xy}$, and the coefficient of the thermal expansion, $\alpha_{x}$, $\alpha_{y}$, $\alpha_{z}$, need to be characterized in advance. Moreover, the Poisson’s ratios should satisfy:

(3a) $$\frac{\nu_{zx}}{E_{z}}=\frac{\nu_{xz}}{E_{x}}$$

(3b) $$\frac{\nu_{xy}}{E_{x}}=\frac{\nu_{yx}}{E_{y}}$$

(3c) $$\frac{\nu_{yz}}{E_{y}}=\frac{\nu_{zy}}{E_{z}}$$

While cooling from a stress-free temperature of $T_{Processing}$ (Figure 1a) to room temperature, $T_{Room}$(Figure 1b), the thermal loading is defined as:

(4) $$\Delta T=T_{Room}-T_{Processing}$$

The material properties might have some nonlinearity, which would call for amendment to Equation (2a–f). During the process, the FR4 in the package accompanies a huge chemical change, which is called cure. The cure is a polymerization process where the monomers or oligomers are three-dimensionally linked. The epoxy resin in the material system is conventionally in the B-stage where the resin is partially cured. In many cases, the cure process impacts the warpage dramatically. Since the cure is an exothermic process that seeks a lower energy state, the molecules become closer to each other. While doing so, a huge volumetric shrinkage is incurred. Sometimes, this can be the main driver of the deformation in the fabrication process. In other words, the thermal strains such as $\alpha_{x}\Delta T$ and $\alpha_{y}\Delta T$ can be comparable to or smaller than the shrinkage by the cure. The problem here is that the cure shrinkage does not appear in any of the above equations. In order for the simulation procedure to accommodate this effect, the processing temperature, $T_{Processing}$, should be adjusted higher.

The method of setting the processing temperature is not straightforward. It requires an inverse parameter estimation method that seeks agreement between simulated and measured deflections at some specified points. The processing temperature of FR4 can be determined by measuring deflections when one side of the double sided PWB is fully decladded. In this case, the simulation is easy thanks to the simplicity of the geometry. The parameter estimation problem is mathematically described as

(5) $$\mathrm{Determine}\;T_{Processing}^{FR4}\;\mathrm{that}\;\mathrm{minimize}\;{\sum_{i=1}^{M}\left[z_{i}(T_{Processing}^{FR4})-{\bar{z}}_{i}\right]}^{2}$$

where *M* is the number of measurement points, $z_{i}$ and ${\bar{z}}_{i}$ are the simulated and measured deflections at *i*-th comparison points, respectively. The way ${\bar{z}}_{i}$ is obtained will be described later in this paper. This method is not very scientific, but is a very reasonable engineering method that allows application to many different material systems. Moreover, complicated nonlinear constitutive equations can be avoided by introduction of $T_{Processing}^{FR4}$. This method has been verified by successfully predicting warpage of double-sided PWBs [10]. It should be noted that the processing temperature by Equation (5) is only applied to the dielectric layers. As for the materials that do not experience any chemical shrinkage such as copper and silicon, the actual processing temperature, $T_{Processing}$, should be imposed without any adjustment. Only for the dielectric layer, $T_{Processing}^{FR4}$ is determined by Equation (5) and applied to Equation (4). Refer to [14] for the detailed procedures.

The deflections are acquired using a laser device on a well-defined surface followed by required transformation to determine $\bar{z_{i}}$. The laser diode position sensor (ILD1401-10, Micro-Epsilon), which has been used in [14], is employed. It is a noncontact sensor that allows reliable measurement. The accuracy of the *x*-*y* stage has been improved from the previous setup [14] by enhancing the rigidity of the frame and the accuracy of the moving guide.

The warpage simulation of the PWB required a quite dense mesh system. The main issue here is that node sharing on the boundary between the copper cladding and the dielectric layer is not easy owing to the pattern asymmetry between the bottom and top layers. Thus, the nodes were not shared between layers and a nonconforming contact mesh was created instead. As a result, the meshing was greatly simplified since each layer could be independently modeled and assembled afterwards. Considering the easiness in handling the contact boundary condition and the versatility in selection of the elements, ANSYS 18.2 was employed to simulate the warpage [24,25]. To implement the nonconforming mesh, the MPC (multipoint constraint) algorithm in ANSYS was utilized. The clad layers were meshed using the three-dimensional shell elements (SHELL63) while the dielectric layers were meshed using a conventional solid element (SOLID45). All the elements were interpolated with linear shape functions, which are suitable for this linear elasticity analysis.

### 2.3. Boundary Condition and Material Properties

The only boundary condition is the fixed displacement and rotation at the center in all directions. The entire domain was thermally loaded in a uniform fashion. It is considered that the PWB is cooled from the processing temperature, 110 °C, which is the curing temperature when it is manufactured, to 25 °C. The dimensions of the considered PWB were 12 mm by 16 mm. The material properties of FR4, Cu, Si are presented in Table 1.

### 2.4. Optimization Model

In this work, the severity of warpage was measured by the difference between the maximum and minimum values in the *Z*-axis after the deformation. Let us call this value the warpage index or the objective, which is defined as:

(6) $$f\left(x\right)=\frac{1}{z_{\max}\left(x\right)-z_{\min}\left(x\right)}$$

where **X** is the discrete input variable, which represents the dummy pattern setup. This will be mathematically defined later. Possible inclusion of minor inaccuracies or uncertainties in material properties cannot overturn the trends and the relative magnitudes of the warpage. Thus, using the material properties in the literature was considered safe.

**Figure 3:**
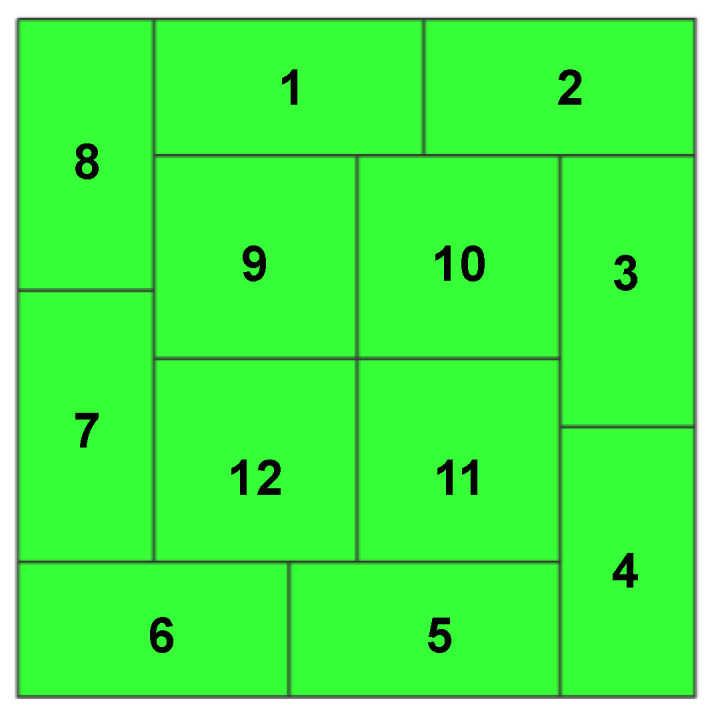
The dummy pattern configurations of the bottom view for the genetic algorithm in the case of Figure 2.

As an exemplary case, consider the PWB in Figure 2. On the bottom side of the PWB, no pattern is present. Thus, the full dummy pattern can be placed and the surface can be divided into the candidate areas in numerous ways. Here, the bottom surface is divided into 12 separate candidate areas as shown in Figure 3. Since each area can have the pattern or not, there are 2^12^ ways of the dummy pattern setting. Each pattern can be represented by 12-dimension binary vector. For example, the full solid pattern can be represented by **x** = (1,1,1,1,1,1,1,1,1,1,1,1) while the no dummy pattern by **x** = (0,0,0,0,0,0,0,0,0,0,0,0). Thus, the optimization problem is reduced to seek the binary number, **x**, that minimizes the warpage. The discrete input variable, **x**, in Equation (1) is a binary vector with *n* dimensions. In general, it can be represented as:

(7) $$x=\left[x_{1},x_{2},\cdots ,x_{n}\right]$$

Note that *n* is equal to the number of dummy pattern regions after the domain discretization.

To save computational costs and time, the number of evaluations should be minimized. However, this optimization cannot be conducted by any gradient-based method since it is a discrete optimization problem. In a gradient-based method, the variables are continuous real numbers. As a result, the gradient evaluation is impossible with the current discrete form. Some discrete systems might be converted to continuous systems, but for the system in this work it is not feasible to do so, since the continuity of the objective function after such a conversion is not achievable. Even when the continuities in both variable and function spaces are guaranteed, global convergence is not easy to achieve since the continuity of the gradient is also required and the solution can be easily trapped into local minima.

Methods based on a neural network can be considered if any deterministic evaluation method is not available. This means that machine learning should be conducted based on experimental data. However, for PWB, it is very costly process and that is why the PWB warpages are simulated. Sometimes, learning by simulation data can be considered if the developed neural net can be reused for other PWB systems. However, such an approach has not been viable so far since each system has individual features and designs which are not easily generalized. Machine learning by simulation data can also work for optimization of a specific PWB problem. This can be achieved by developing a neural network that works only for a specific problem, and then applying that neural net to the same problem. However, this could be more costly than or similar to the exhaustive search.

Thus, the current problem is best suited to the optimization by the genetic algorithm (GA). The candidate area can be copper-clad or not clad during the optimization process using the GA [27]. The GA has a long history, but still it is not a simple matter to apply this approach to a specific problem since definition of the optimization problem would dominate feasibility of the solution. This work proposes a GA method that is customized to the warpage optimization problem. To be a practical method, the method should return the solution in an affordable time range and should be able to accommodate the industry conventions in a flexible way. By doing so, the possible trial and error can be minimized.

### 2.5. Genetic Algorithm

Let us introduce the GA first. It is a non-gradient-based general method for optimization problems. When a complex problem has to be solved, the traditional gradient-based method might not provide the solutions in the desired time line. The GA has proven that it can deal with such complex problems efficiently [28,29]. It has yielded many quality solutions for optimization problems successfully realizing the evolutionary algorithm. This nature-inspired algorithm is mainly comprised of crossover and mutation. To execute the algorithm, input variables should be represented as the chromosomes with several alleles. Alleles are the minimum unit that carries the information that can affect the objective of the optimization problem. An allele in the GA can hold a value of either 0 or 1, which is not expressed in a pair form as in biological alleles. A small group of alleles constitute a gene. Notice that the alleles in the same gene should be handled and interpreted together. Then, a collection of genes constitute a chromosome.

In the configuration shown in Figure 3 and other problems with a dummy pattern setup, a chromosome with a single gene can represent the problem suitably. Therefore, this work considers a chromosome comprised of several alleles. In other words, the binary vector, **x**, in Equation (2) represents the chromosome and each binary component in **x**, $x_{i}$ does the *i*-th allele. In this work, the optimization problem is defined as:

(8) Determine x that minimizes the fitness function f(x)

Since **x** directly denotes a specific dummy pattern setup, the phenotype and genotype are identical in this optimization problem. Since the size of the search space is as large as 2*^n^*, an exhaustive search cannot achieve the goal when *n* is large. In GA, the alleles in **x**, are recombined by the crossover. This work selects so-called uniform crossover where the offspring chromosomes are created by swapping the binary vectors divided by a randomly selected single point, *c*, between 1 and n−1. Suppose two best parents $x^{p1}$ and $x^{p2}$ from the current population, which are expressed as [27]

(9a) $$x^{p1}=\left[x_{1}^{p1},x_{2}^{p1},\cdots ,x_{n}^{p1}\right]$$

(9b) $$x^{p2}=\left[x_{1}^{p2},x_{2}^{p2},\cdots ,x_{n}^{p2}\right]$$

Then, two offspring chromosomes are generated by exchanging the alleles divided by the point *c*. As a result, we have,

(10a) $$x^{o1}=\left[x_{1}^{p1},x_{2}^{p1},\cdots ,x_{c}^{p1},x_{c+1}^{p2},\cdots ,x_{n}^{p2}\right]$$

(10b) $$x^{o2}=\left[x_{1}^{p2},x_{2}^{p2},\cdots ,x_{c}^{p2},x_{c+1}^{p1},\cdots ,x_{n}^{p1}\right]$$

By doing this, the offspring can inherit the best properties from the parents as in nature.

**Figure 4:**
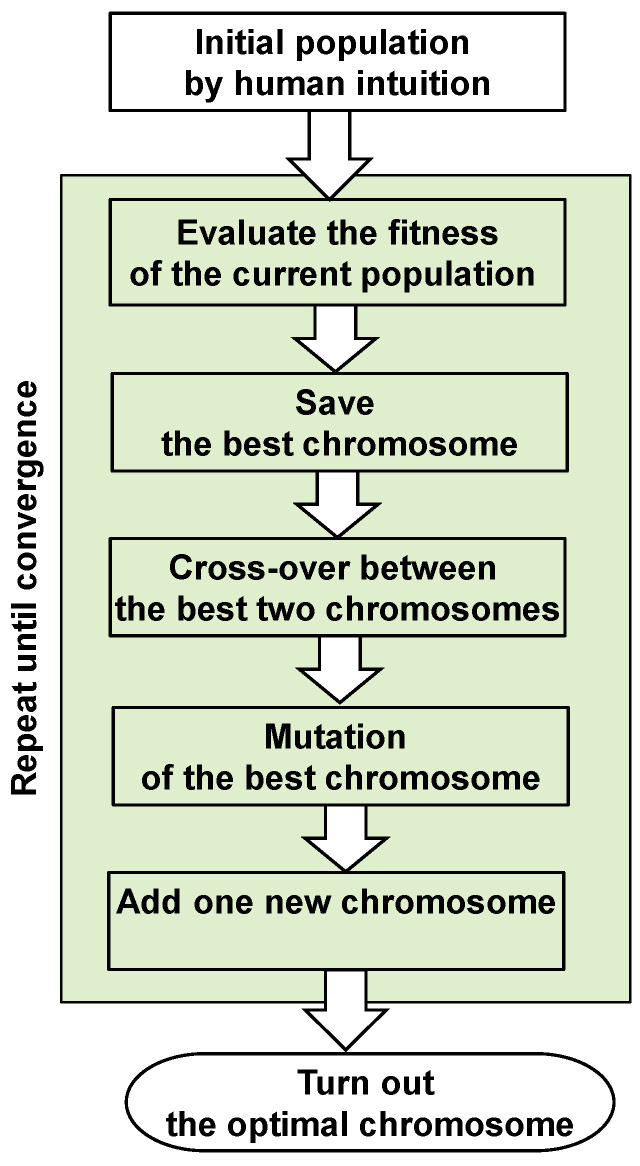
The algorithmic flow to the optimal dummy pattern by the genetic algorithm.

The crossover operation works like a random search on a global space. To seek a locally fitter solution, a small number of alleles needs to be switched by random or systematic mutations. In the mutation process, a selected set of alleles are changed from their current values. The GA procedures including the crossover and mutation are shown in Figure 4. In this work, an initial population with five individual chromosomes is generated intuitively by a human agent. Then, the finesses of the entire population are evaluated by calculating the difference between maximum and minimum out-of-plane displacements from the result of the warpage simulations by Equation (1). Then, the following four steps are repeated until the fitness solution does not improve:

- Select the two best parents among the existing individual chromosomes.
- Perform crossover between the two best individual chromosomes by the uniform crossover by a random process.
- Perform mutation to the best individual and add the result to the next population.
- Add one purely new random chromosome to the population.

### 2.6. The Human-Based Genetic Algorithm

When many alleles are set, say 50, the number of iterations can be very high. Especially for this sort of problem where the function evaluation is costly, it is necessary to reduce the number to complete the design in time. Human experience in the specific problem would help reach the solution rapidly. The human-based genetic algorithm (HBGA) is employed to utilize the experiences of design engineers who resolved the warpage problems by trial and error [29,30]. In HBGA, the recombination process and the fitness function evaluation can be outsourced to human agents. The recombination includes crossover and mutations of the chromosomes.

In this work, only mutation is outsourced to a human agent who learned how the PWB warpage can be alleviated from their own experiences as a PWB design engineer. The specific mutation method that the design engineers used are not fully described here, but there are two main factors that should be mentioned. First, the imbalance in the remaining amount of copper should be rectified considering the current snapshot of the warpage. Second, mutation in the outer layer and the location far from the fixed center should be considered first. The HBGA can also utilize the ideas from cumulative knowledge obtained while solving similar optimization problems. In an earlier work, the HBGA was employed to control the warpage of a double sided PWB [9]. Therein, random and human-agent mutations were alternated. This required intensive human engagement and communications, which are inherently costly. In this work the human agent step is improved by exploitation of a priori information. The mutation process is designed as follows:

- The mutation is conducted under a constraint set by the a priori information. If this configuration reduces the warpage, finish the mutation operation.
- Try a random mutation. If this configuration reduces the warpage, finish the mutation operation.
- Otherwise, try an attended operation of mutation by a human agent.

The a priori information should be described by an experienced PWB engineer who can play the role of a human agent. It should be described how a human agent would make a decision. It does not have to be very accurate since it is a process of trial and error, but it should comprise logical statements that can be implemented in a computer code. Although this does not have to be based on mechanics, eventually it causes a change in mechanical deformation. The desired conditions have been described and they are imposed with prescribed priority. The simplest thing is to evenly distribute the remaining copper all over the area resulting in a uniform distribution of copper at a macro level, but the copper pattern generally does not allow that. In this development, the status of the copper distribution has not been intentionally pursued. Because it is sometimes not viable to do so, the pattern should be selected to enhance the rigidity that resists against the warpage or reverses it. This requires detailed information regarding the simulation in addition to the objective function defined by the warpage index. The magnitude of the strain at the center of each allele is evaluated and ranked. If the highest rank allele is on (1), its value will be changed to off (0). In the case when it is on, the closest allele currently off is set on. The distance between alleles needs to be calculated and recorded prior to the optimization in order not to repeat the same calculations. The automated mutation process is conducted as follows:

- First, retrieve the simulation result.
- Second, determine the off allele with the highest rank, A1.
- Third, determine the on allele with the lowest rank with 1, A2.
- Decide whether to reverse both, or either A1 or A2, based on the random number generation, independently.

Retrieval of the strain magnitude data requires some plumbing codes that scan the text results file to obtain those values where designated in advance. While it is trial and error, it has been found that this approach can contribute to reducing the number of human engagements.

When the human agent is engaged, very rich information can be provided to the agent. However, an expert who has enough industry experience in controlling the PWB warpage is very rare. Thus, to train human agents, the principle should be described in detail, including how to exploit the simulation results. This does not have to be defined as a procedural protocol since it resorts to the intuition of the agent. Thus, simply the principles are stated to allow flexibility as follows:

- Try to set alleles at sites where copper looks deficient.
- Set alleles on the opposite side to the center of the radius. This applies also when a saddle occurs.
- On the edges, if deflection is higher than the other ones, set alleles on nearby.

### 2.7. Implementation

As stated earlier, the warpage simulation was conducted using ANSYS 18.2. It also provided a tool for pattern homogenization, but this work simulated the warpage for the geometry as is. The most challenging part was how to update the geometry while simulating. It should be somehow possible to update the geometry and mesh it repeatedly during runtime, but it is very computationally expensive as well as implementationally complicated. To realize this capability with a fixed geometry, it is advantageous to control the material properties.

The essential point is how to practically remove the dummy pattern by changing the material properties. As a matter of course, the mechanical properties have to be lowered. Thus, the Young’s and shear moduli have to be overwritten. In the candidate area, when the allele value is 0, the Young’s modulus, *E*, is set as 1% of the copper value. Note that imposition of 0 Pa to *E* causes numerical instability. Simply by changing this value, the dummy pattern effect can be fully simulated. To do this, each candidate area should be treated as a separate subdomain that allows individual imposition of the material properties. Once the input file is created, the position of the specific material properties in the file stream can be located and the modification can be easily done with the text handling functions.

To realize the optimization process, a VBA (Microsoft Visual Basic for Applications) code in Microsoft Excel was written. The VBA code modified the ANSYS ADPL (the ANSYS Parametric Design Language) file directly. Then, it executed the batch file to obtain the ANSYS result file. The VBA code read the maximum and minimum deflections to determine the warpage index. To read and write to the ADPL file, the VBA functions for the text operations were intensively utilized [31]. Whenever the human engagement was required, the excel VBA paused the run and waited for the input, popping up a dialog box. To determine the crossover point, a worksheet function of Excel for random number generation was repeatedly called. Note that when user intervention was unnecessary, a commercial optimization tool such as VisualDoc could be put into use.

## 3. Results

### 3.1. Double Sided Case

As aforementioned, the copper cladding was meshed by three node triangular shell elements while the dielectric layer by the 20 node brick elements. A total of 16,021 elements and 21,382 nodes were employed in the discretization. In the test run, the simulation finished in approximately 52 s on a PC (with an Intel Core i7-4880K 3.5GHz processor) built by the authors. The thicknesses of the copper cladding and the dielectric layer were 25 μm and 60 μm, respectively. To check the feasibility of the optimization, two extreme cases were tested first. When no dummy pattern was employed, the warpage index was 0.277 mm. With all dummy patterns the value was 0.152 mm. With randomly generated chromosomes, the warpage index was mostly smaller than 0.2 mm. Thus, there is great possibility of having a feasible as well as nontrivial solution in this optimization. For the processing temperature of 110 °C, $T_{Processing}^{FR4}$ was found as 207.3 °C by Equation (5).

**Figure 5:**
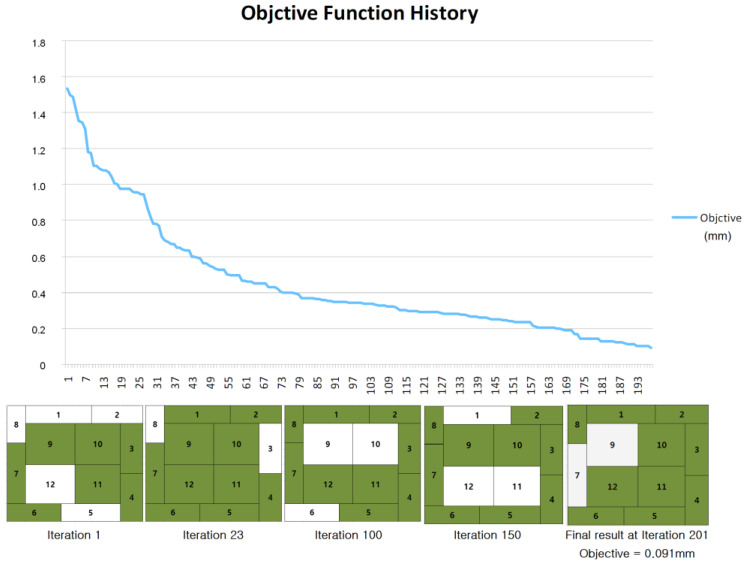
Optimization using the genetic algorithm.

**Figure 6:**
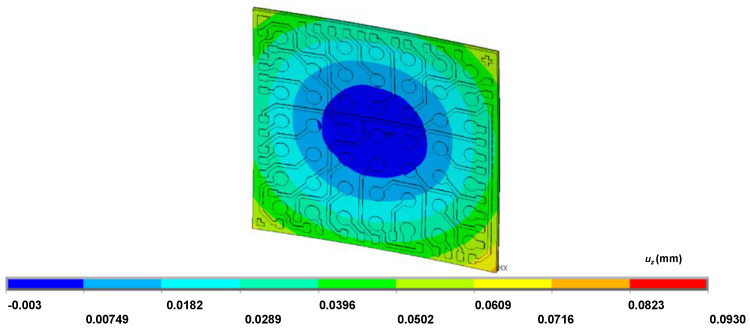
The simulated result with the full dummy pattern.

We conducted the HBGA optimization as described in the previous section. A total of 201 iterations were conducted and the optimal solution of 111111010111_(2)_ was reached. The change of the objective function and the dummy pattern while the optimization procedure is shown Figure 5. A rough but monotonic decrease is noticeable there. Figure 6 shows the final simulation result reached by the optimization.

### 3.2. Multilayer Case

**Figure 7:**
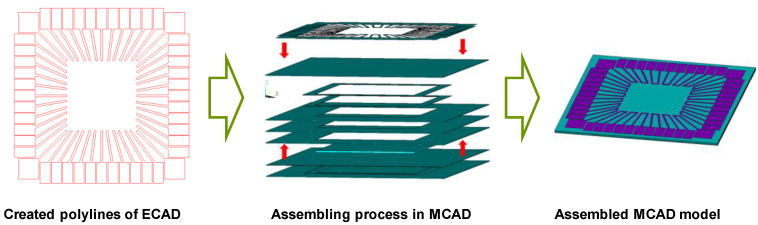
Application to a multilayer PWB case.

**Figure 8:**
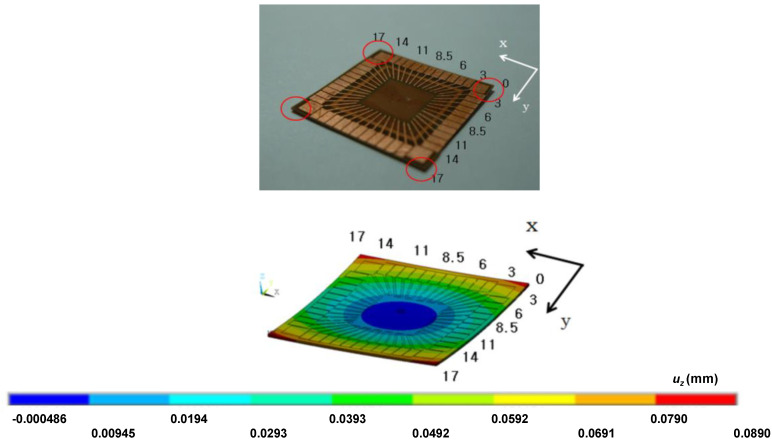
Comparison of the simulated result and the fabricated PWB.

**Figure 9:**
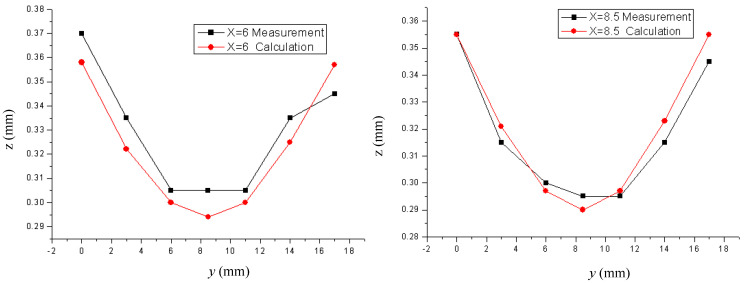
The experimental and numerical *z* position values along *x* = 6 mm (**left**) and *x* = 8.5 mm (**right**).

Consider a multilayered PWB, which is designed to be used for a chip-in-board (CIB) package. The structure and outline of the package is presented in Figure 7, which shows how the ECAD model can be reconstructed to the MCAD one. Since the chip is embedded in the dielectric layer, no patterns are present in the central region. Figure 8 compares the actual package built and the simulated deformation. Each corner is elevated from the bottom because of the smiling warpage mode. As can be seen in the figure, the simulation mimics such a deformation quite well. Recall that the processing temperature of FR4 was set by the inverse parameter estimation using experimental measurement data [32,33]. To quantitatively guarantee the accuracy of the simulated deformation, the experimentally measured values are compared with the simulated results. Figure 9 shows a comparison along the *y*-direction for *x* = 6 mm and *x* = 8.5 mm. The comparisons give good agreements between experiments and simulations. There could be other methods for evaluating the warpage [34], but the measurement method using the CCD position sensor has been employed in this work with consideration of availability and reliability [14].

**Figure 10:**
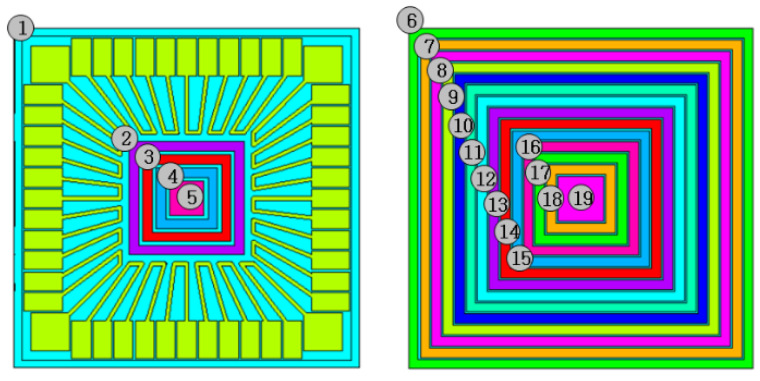
Dummy pattern setup for optimization by the HBGA.

**Figure 11:**
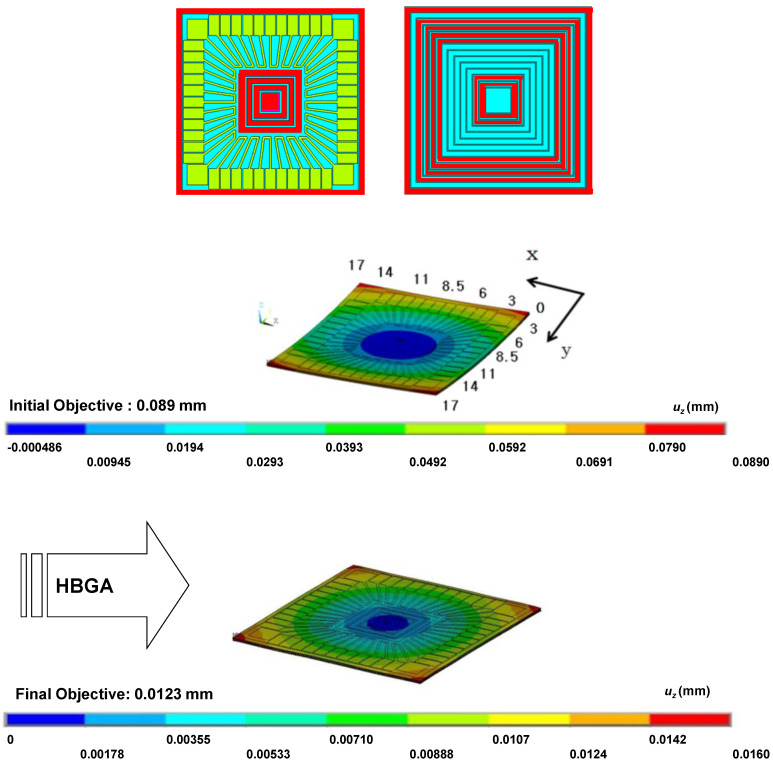
Optimized dummy pattern by the HBGA.

The candidate areas for the dummy pattern are shown in Figure 10. A total of 19 locations are prepared for the dummy pattern. Since the number of possible combinations is 2^19^ = 524,288, an exhaustive search is not viable. Moreover, in this case, the number of nodes is 82,370 and the single evaluation by simulation requires 329 s. In the optimization problems based on numerical simulations, the simulation time for a single run is a critical factor for the success of computation [35]. The HBGA method reached an optimum after 312 iterations. Figure 11 shows the optimal dummy pattern giving a simulation result with 74.2% of the warpage reduction.

## 4. Conclusions

The method of dummy pattern setting has been proposed to control the warpage prevalent in fabricated PWBs. To set the dummy pattern, the candidate areas have to be determined first. Whether to set the pattern in each area or not is decided while conducting an optimization that minimizes the maximum deflection. In this optimization, the deflection has been evaluated by structural simulation. The simulation was conducted using ANSYS 18.2 and the optimization was realized by the human-based genetic algorithm. PWB warpage was simulated without any pattern approximation such as homogenization to avoid any possible entrainment of error. The whole procedure was implemented in a Microsoft Excel spreadsheet with the VBA code. It modified the ANSYS input file and read the output file. The mutation, crossover and human interactions were also implemented using the VBA. Human engagement in this optimization was allowed only for mutations. This operation was done by a human agent who knew how to change the design to alleviate the warpage. This work has also proposed how to partly automate the mutation process using prior information. Moreover, the rules that the human agent should follow have been described.

This work has applied the proposed method to two different cases. In the first case, a double-sided PWB with single dielectric layer has been analyzed. Then, in the second case, a multilayered PWB for the chip-in-board package has been optimized and the warpage could be reduced by 74.2%. As shown in the test cases, the proposed optimization method based on the structural simulation and the human-based genetic algorithm can be applied to PWB dummy pattern designs.

## Figures and Tables

**Table 1:** Material properties for package substrate [14,26].

	Dielectric FR4	Cu	Si
*E_x_*	11.8 GPa	103.4 GPa	129 GPa
*E_y_*	11.8 GPa	103.4 GPa	129 GPa
*E_z_*	7.5 GPa	103.4 GPa	129 GPa
*ν_xy_*	0.27	0.3	0.28
*ν_yz_*	0.22	0.3	0.28
*ν_zx_*	0.22	0.3	0.28
*G_xy_*	3.0 GPa	39.71 GPa	64 GPa
*G_yz_*	3.9 GPa	39.71 GPa	64 GPa
*G_zx_*	3.9 GPa	39.71 GPa	64 GPa
*α_x_*	11 × 10^−6^/°C	17.6 × 10^−6^/°C	26 × 10^−6^/°C
*α_y_*	11 × 10^−6^/°C	17.6 × 10^−6^/°C	26 × 10^−6^/°C
*α_z_*	16 × 10^−6^/°C	17.6 × 10^−6^/°C	26 × 10^−6^/°C
